# Kentucky State Dental Association

**Published:** 1879-09

**Authors:** Chas. E. Dunn


### Proceedings of Societies.

### KENTUCKY STATE DENTAL ASSOCIATION, HELD IN LOUISVILLE, JUNE 3, 4, AND 5, 1879.

### DISCUSSIONS AT THE NINTH ANNUAL MEETING.

*Reported by Dr. Chas. E. Dunn.*

MORNING SESSION.—SECOND DAY.

"Secondary Dentine, Osseous Pulps, Exostosis, Influence and Effects of Dead Teeth."

Dr. Rawls, of the committee, had no written report in regard to secondary dentine on the subject, there is nothing particularly new; its pathology is perfectly clear to some, they claim that plasma is thrown out from the pulp, which gradually hardens; while others claim that it depends on secondary causes, such as any undue inflammatory condition. Have nothing further to say on this subject, at present. Would like to have it pass in to the hands of those that have given the subject more study.

Dr. J. Taft—Will you please define secondary dentine?

Dr. Rawls—It explains itself; it is a reformation of something that had once been formed. How this is done, I leave Prof. Taft to explain.

Dr. Kidd—As far as I have been able to ascertain, it is something like true bone. We find it in teeth, outside of the pulp itself, I don't think any full explanation can be given of it. A certain physician of our town, seems to think it a deposit like tartar; the fibers and tubuli throw off a substance, which being acted on by the fluids of the mouth, hardens. We know the oral secretions will act on certain substances so as to cause them to solidify.

Dr. Herriott—Would like to ask Dr. Rawls what is the condition in teeth worn down by excessive mastication. We see teeth of this kind, with the grinding surface exceedingly hard. Is that secondary dentine?

Dr. Rawls—In wearing down of the grinding surface of the teeth, dentine breaks down, and is impacted in the tubuli. It is nature acting to protect and repair the injured or exposed part. We see this in capping exposed pulps; the pulp recedes, throws out plasma, covering itself, which gradually solidifies, the pulp continuing to recede as the solidification takes place.

Dr. Kidd—How does Dr. Rawls account for the receding of the pulp?

Dr. Rawls—By some irritating process on the pulp, and without any breaking down, it is a physio-pathological action.

Dr. Herriott—Nature don't do that kind of awkward thing; making it necessary for the pulp to get out of the way. I oelieve it is as thoroughly organized as true dentine.

Dr. Rawls—The distribution of nutrition must be carried on. Cut this off, and death is the result. We can have breaking down of dentine, filling up of tubules, and hardening of substance. How do you account for wearing down of points of molars? how do you account for this condition, without vitality to prevent further breaking down? I cannot think this action is throughout from the pulp; it is simply breaking down of the dentine, and the impacting of it in the tubuli.

Dr. Kidd—Stricker says there are tubuli in secondary dentine, but they don't run clear through to the end; these tubuli are filled with a fluid substance, and this bony formation we are speaking of, comes from this fluid.

Dr. H. A. Smith—I suggest these subjects of physiology, pathology and histology be discussed together.

Dr. Peabody—Dr. Smith's suggestion is a good one, I don't want to take up the time of the association discussing this question, but would like more information as to the physiological action of secondary dentine. It struck me while this question was being debated, that this throwing out of secondary dentine, where the pulp shrinks, and osseous matter takes it place, was something similar to a cicatrix, or the reparative process. When a tree is wounded, or a knot on it formed, the part becomes sufficiently organized to permit nutrition, yet not organized as in the first formation.

I saw a case a few weeks ago, which caused me a good deal of thought, and I am still unable to account for it. The patient is a little girl, about eight years old, perfectly healthy, never having had any of the diseases incident to childhood. No scarlet fever, measles, chicken pox, or any cutaneous eruption. The temporary teeth were perfectly formed, but the permanent incisors, both above and below, while being perfect in their formation from the gum for one-half their length, were from the centre to their cutting edges, pitted all over in regular lines, the inner plates of enamel with the dentine being absent, and in the lower teeth, the outer plates so thin, it seemed as though they must break if used. I hardly dared to touch them, they are so frail. Had the child been at all sick during the time those teeth were undergoing calcification, it might be accounted for, as it is, I am at a loss for an explanation.

Dr. Rawls—It is more of a malformation than a pathalogical condition.

Dr. Doyle—Would like to ask Dr. Peabody if there is any caries in these teeth?

Dr. Peabody—None whatever.

Dr. Doyle—I had a case somewhat similar. except that there was caries in the pits, it was the case of a young lady, about sixteen years old, her father wanted the teeth extracted, but I would not consent. After the three molars were erupted, then began a wasting of the enamel all over the crown, I did all I could to save them, but could not succeed. I tried polishing, but found the softening extended through the entire substance. Extracted the teeth last Monday, they were soft, could cut them away anywhere with an excavator; every tooth in the mouth was affected, upper and lower. The lady is now about twenty years old, had scarlet fever when about five years of age.

Dr. H. A. Smith—I had a case similar in nearly all respects—except the central incisors—the cutting edges or projecting portion, had a pinched appearance. It occurred to me, that these portions had no dentine, but were formed entirely of enamel. The child was very delicate. Several children in the family all healthy but this one. In this case there was excess in formation of enamel.

Dr. Peabody—Could anything be done to relieve by systemic treatment?

Dr. Smith—I think not; the harm is done, and is beyond our reach. In my case, I cut the affected teeth down, so as to avoid striking on the other teeth, thus relieving them somewhat by rest.

Dr. H. A. Smith—Let me say a word on the practical phase of this subject. Suppose you have exposed pulp, and the odontoblasts are destroyed with creosote, carbolic acid, or something of the kind, you could not have the matter thrown out for this secondary formation. The function of the odontoblasts, according to the latest theory, is to produce new matter. Age would make a difference, as there is of course, more vitality at sixteen years than at sixty.

Dr. Rawls—This secondnry tissue is organized, otherwise nutrition would be shut off. It may have, as Dr. Smith said, interstitial supply, if this supply is cut of by deposit, where are your canals? You can't have the supply pass through dead matter. Healthy nutrition does not pass through dead tissue.

Dr. Herriot—I desire to relate a case which occurred in our town just before I left home. It is the case of premature birth of a child—seven months gestation—with an entire set of teeth. If possible, I will procure a cast of the head, and present it to the association.

Dr. Kidd—I have a patient fifteen years old, who is perfectly healthy, but has only two permanent teeth. Would like to hear the opinion of the members, as to the cause of this tardy development.

### MORNING SESSION.—SECOND DAY.

Dr. Hooper, of the committee on physiology, read an interesting paper on "The Provisions of Nature, for the Protection of Exposed Pulp."

Dr. Taft—In regard to the subject on the programme, "Action and Effects of Dead Teeth," we must understand what is meant by "dead teeth." Some say, "when the pulp is dead, the tooth is dead." In young persons, especially after the destruction of the pulp, the contents of the tubuli, undergo considerable change, while the periosteum, in any case, remains, the cementum will retain its vitality. Let the cementum loose its vitality, and the surrounding parts will no longer tolerate the dead member. In young persons, it is far more important to preserve the pulp, for then periosteal trouble is more liable to occur, and in their case, the effects and results of dead teeth on the system, are more serious. Subject passed.

Dr. Edwards, of the committee on anatomy, said: The question whether "nerves reunite in replanting," is answered yea or nay, according to circumstances. In amputation of a finger, and replacement, some of the nerves may unite, while some do not, but that is a different case from a single minute nerve in a tooth, with bone surroundings; strangulation would frequently prevent its uniting. Dental literature is very meager on the subject. I have heard and read of some reported cases, but have never seen any. The only cases to test the question is where a tooth is knocked out by accidental violence.

Dr. Herriott—Dr. Edwards has struck the point exactly. In most cases of replanting, the nerve is dead at the time. I had a case where the tooth was knocked out by accident, I replaced it immediately, and after a short time it became perfectly firm. Four years afterwards, the patient returned with exposed nerve, in cavity of same tooth, I reported the case to the American Dental Association, and was hissed, I felt very mean, but Dr. K———, came to my assistance, and said "nerves would reunite."

Dr. Edwards—Just thought of case in my own practice; in trying to remove a first molar, the patient's struggles caused me to extract the second bicuspid, I replaced the latter at once, never saw the patient afterward, but learned from other members of the family, that after some little soreness, the tooth was all right, and gave no further trouble. Some say nerves contract after amputation, that is not always the case, I have seen cases where the nerves protruded.

Dr. Rawls—Medical literature ought to settle this question. In cases of resection of a nerve for neuralgia, for a while there would be no pain, but after some years, the pain would return, showing the nerve had reunited, and showing the possibility of nerves in any part uniting. Remember hearing Dr. Geo. Watt, report the case of a boy having a tooth knocked out while coasting, the tooth was lost in the snow for some time, and when found, was completely frozen, the Doctor, after thawing it successfully in cold tepid and warm water, replanted, and since then, to all the known tests, it has responded as a living tooth. I once had a case similar to that of Dr. Edwards. In extracting a decayed, second lower bicuspid, which impinged on the first bicuspid, the latter popped out clear across the room, I replaced it, saw the patient six months afterwards, and the tooth responded to every test. There is no doubt in my mind that nerves reunite, but whether we are justified in risking the operation, except in such cases of accident, is another question.

Dr. Herriott—I must say that I endorse Dr. Rawls, and would advise all when replanting, except in cases of accident, to fill the root to the apex.

Dr. Taft—I have seen very few such cases. Even in the most favorable cases, when a tooth is removed, there is rupture, not only of the nerve, but also of the artery and vein, and connecting tissue. Now if the mouths of these different broken parts be brought into exact opposition, nerve to nerve, vein to vein, etc., they might possibly unite. The fact that nerves reunite in favorable conditions, shows us the possibilities of nature, but I do not think the operation advisable in practice, except in accidental removal, would much rather fill root canal before replanting.

SECOND DAY—AFTEROON SESSION.

Subject, "What are the Conditions of the Alveolar Process, When There is Abscess on the Tooth?"

Dr. Taft—The conditions depend on the tissue involved, whether it has an external opening, and whether the periosteum is much affected, the alveolus may retain its position; it sometimes recedes, and serves as a pocket for pus, necrosis may take place and exfoliation. In other cases the osseous debris may be dissolved down, and carried off in the fluids, and almost any variety of conditions may arise in an extensive abscess.

Dr. H. A. Smith--Suppose you had a chronic case of abscess? Could not cure, had been running on for years, whence comes that enormous quantity of pus? Is there any destruction of the surrounding tissues?

Dr. Taft—In chronic cases there is little or no destruction, in active cases there is rapid destruction.

Dr: Edwards—What is it that causes swelling in cases of abscess?

Dr. Taft—It is due to exudation from the blood, infiltration of the tissue outside of the vessels with plasma, and then at a later stage with pus.

Dr. Peabody--About a month or so ago, almost every case of inflamed nerve, in my practice, was accompanied by swelling, One case where nerve was dead, tooth had been filled, no pus, but swelling, I attempted to treat, but could not touch it without causing swelling.

Dr. Taft--I have no doubt that it was an atmospheric influence that affected your patients, probable malaria.

Dr. Edwards--About the time Dr. Peabody speaks of, we had very sudden and severe changes in the weather, I had several cases affected in that way. I never permanently fill devitalized teeth, when severe atmosphere changes are going on.

Dr. Herriott—There is no doubt this trouble comes from malaria. I have found where there was dead pulp and swelling, the best treatment to be, to fill with salicylic acid and let it remain several days.

Dr. H. A. Smith—I think the atmospheric conditions are not always the cause. It would be well to look to the hygienic condition of our offices and instruments. A lady called on me to have a tooth extracted, I advised her to have it treated, I used a broach, and that tooth cost her sixty dollars.

I think these conditions could often be traced to local causes in our offices.

Dr. Peabody—I apprehend if Dr. Smith had investigated a little further, he would have found that this broach had carried some poisonous matter through the foramen, and caused disease. These severe changes of the weather will cause this slowness in curing abscess, I sometimes advise hot foot baths, etc., with beneficial results. Subject passed.

"The Different Agents that Produce Disintegration of Enamel and Dentine, and their Mode of Action."

Dr. Cassidy—Some experiments, bearing on this subject, that should have been completed in time to report the results to this meeting, are yet in a state of gestation, so that I am unable to present a written report. Some thoughts however in this connection, may be stated, and I hope you will not consider me dogmatic, while trying to be brief. The great difficulty of arriving at a correct, demonstrated knowledge of the exciting specific cause of tooth destruction in each case as presented to us, is fully appreciated by any one who has experimented in this direction, indeed an exact recognition of the chemical or physical nature of those things which induce diseases, is the great unattainable of medical science. Most of us take it for granted, that it is invariably an acid, which in each case, causes caries of the teeth; we have been told so by those who ought to know, and therefore we believe, so with these pre-conceived opinions, we experiment.

One experimenter selects a number of extracted teeth, covers them with some impermeable substance, like sealing wax, except a small point of surface, which he leaves exposed for months, perhaps years, to the action of some given acid; another immerses teeth, having different kinds of fillings in them, in a certain well known solvent, and notes the relative rapidity in the destruction of each; and another prepares a fermenting mixture of some kind, keeps it at a given temperature, and subjects for a long time a number of teeth to its destroying influence; and finally they publish their discoveries, and we, who may be comparatively ignorant, must smile at their many mistaken conclusions. These experiments, crude, and necessarily tedious, only serve as a good foundation upon which to build the yet future science of the etiology of dental caries. It is encouraging to know that even a few dentists are experimenting in this pecuniarily unprofitable direction, teaching us by modest example that the love of science is, at times, above that of money; and although my respect for them is beyond expression, I feel constrained to say, that at least two of the classes of the experiments I have mentioned, have no practical analogy in the mouth. How many of you here, have ever found saliva of a pronounced acid reaction? My own observation has been, that such cases are extremely rare, not the one-tenth of one per cent, even in the most woeful cases of decay. I am not at all surprised at this, for if you ever find the teeth bathed in acid saliva, you will not find characteristic caries, but the teeth have a sort of abraded surface, and on the living ones, marked sensibility, conditions plainly due to the general equality of the surrounding liquid, and the near equality in the structure of the teeth. Now such is also the case—minus sensibility—when extracted teeth are immersed in a given acid; general surface decomposition takes place, not the formation of caries, unless, like one of the experiments I referred to, we seal the teeth hermetically, except a little place, which of course soon burrows into a beautiful cavity. Vitality has nothing to do in resisting the first attack of caries, the resisting power resides in the good structure of the tooth, but as the solvent encroaches on the pulp, then vitality is generally sufficient to neutralize for a considerable time the chemical action. There is no doubt in my mind, that the solvent molecules which cause caries—aside from possible galvanic development—are due to the different kinds of fermentation, but the different characteristic acids, either mineral or organic, which would be produced en masse, by undisturbed fermentation in quantity, need not manifest their acidity at all previous to attacking a tooth; one or two examples of this fact will suffice, thus, where nitrogen is involved in the formation, or putrefaction, if you like it better, the presence of the well known alkali, ammonia, is antecedent to the formation of nitric acid, so that you test for an acid, and at the very threshold you find an alkali. Again the electro negative radicles of some of the acids can do the mischief as they develope, and they are not acids, they are simply the radicles of acids; thus chlorine, in the ratio of its nascent energy, will unite with lime salts, whether set free from hydrogen, as in hydrochloric acid, or from union with any other positive element; and chlorine is not an acid, it is only the electro-negative of the chlorine acids. These radicles as they develop, may be actually at work on a tooth, at the very moment we are testing for acids; and their work is molecular, in situ; that is the solvent, whether it be a radicle, or a full grown acid, does not wait after the birth of its first molecule for reinforcements, and then go exploring through the mouth to find an enemy; it attacks at once, at the very instant and point of its molecular development, or otherwise it is last, neutralized by the basic molecules in the moving liquid.

It is of course, unnecessary for me to say anything about the important structural defects in enamel and dentine, predisposing to disease; neither perhaps, should I impose further on your time just now, by entering in detail on the chemical changes that occur, when organized substances are passing into simpler forms, in the many varieties of the process, we call fermentation, for lack of a better term, we should remember however, that the tendency is to terminate the process with the production of the acid that corresponds to the type of the fermentation. For instance, dissolved starchy substances at the animal temperature, is converted by saccharine ferment, into glucose;* and glucose by the proper ferment, is changed into alcohol and carbonic oxide, the alcohol by absorption of oxygen, from the atmosphere or elswhere, is

* Starch. Water. Glucose.

$$C6.\ H10.\ O5. \times H2.\ O. = C6.\ H12.\ O6.$$

Glucose. Alcohol. Carbonic oxide

$$C6.\ H12.\ O6. = 2\ C2.\ H6.\ O. \times 2\ C2.\ O.$$

Alcohol. Aldehyde. Water.

$$C2.\ H6.\ O. \times O = C2.\ H4.\ O. \times H2.\ O.$$

converted into aldehyde and water, and aldehyde, by absorption of oxygen, is changed into acetic acid,* at which point acetous fermentation ceases. But glucose, in fermenting, does always have to call on alcohol and oxygen, in order to reach a typical acid; by the aid of a lactous ferment, one molecule of glucose by the mere transposition of its constituent atoms, is changed at once into two molecules of lactic acid, and one molecule of the sugar of milk, by the simple assumption of a molecule of water, is converted into four molecules of lactic acid.†

That there are sugar forming ferments, in saliva, as well as in pancreatic juice, there can be no doubt, they can be easily separated by dilute phosphoric acid, neutralized with lime water, precipitated with alcohol, and dried at common temperatures. Such ferments are never absent from saliva. Is it therefore any wonder that the destructive typical acids of the glucoses should be readily formed in the mouth, where all the circumstances necessary to the process of fermentation are known to be present? Their prevention however, is something for the future to attain.

Dr. H. A Smith—I have made some tests in the college infirmary last session, and found acid reaction in some of the cavities of decay. Dr. Black, of Illinois, says it is well known that caries seldom occurs on the lingual surfaces of teeth. The friction of the tongue prevents adhesion of fermenting substances, while at certain other points destruction rapidly takes place, because there is adhesion there. I think Dr. Cassidy is right in saying that saliva is seldom acid; were it so they would all tend to suffer nearly alike, all their surfaces being bathed in the same saliva.

Dr. Rawls—I have seen many cases of rapid caries, which seemed due to common salt. Such of my patients, in reply to

* Aldehyde. Acetic acid.

C2. H4. O. × O = C2. H4. O2.

† Glucose. Lactic Acid.

C6. H12. O6. = 2 C3. H6. O3.

Milk Sugar. Water. Lactic Acid.

C12. H22. O11. × H2. O. = 4 C3. H6. O3.

questions, would ask me how I knew they were great eaters of salt. Will Dr. Cassidy inform us how this substance acts?

Dr. Cassidy—Common salt can act upon teeth in many ways; that is, through the agency of many things, its electro-negative element, chlorine, can be set free; and nascent chlorine can decompose calcium carbonate—forming soluble calcium chloride—in the ratio of its energy of liberation, just as well when freed from union with sodium as from hydrogen. Hydrochloric acid indeed re-acts on teeth in the same way, by giving up its chlorine. Sometimes it is a negative radicle, like sulphuric acid, which takes away the sodium of salt, or nascent hydrogen produced in fermentation, and various other ways, can form hydrochloric acid by taking chlorine from salt, or a solution of common salt itself, will dissolve a tooth, but, all these cases depend for final action on the tooth, upon the chlorine of the salt.

Dr. A. Wilkes Smith—I would like to know something of the green stain on the teeth of children; would like to hear Dr. Cassidy's opinion on the subject.

Dr. Cassidy—The green stain that Dr. Smith refers to, is evidently a product of some kind of fermentation, and is found chiefly on the anterior teeth of children. Let us begin at the beginning.

The portion of food that has a decided glutinous attachment for enamel, is the starchy debris, as it agglutinates on a part it affords an excellent surface for the addition of more. Now such debris begins to ferment, the result in the majority of cases, is either the lactous or acetous kind; on the anterior teeth, on account of the easy access of air, it would tend to the latter form. Fermentation of all kinds is always accompanied by development of organisms of the fungus class, and the fungus that accompanies acetous fermentation is greenish in color, but whether the growth of this deposit is a cause, or consequence, of acetous fermentation, I can not say. I can only say that my microscope reveals no difference in the fungus of well known acetous fermentation, and that of the green deposit on the teeth. The enamel of such is often softened, sometimes a cavity formed, showing the development of some solvent, but acetic acid, is fortunately comparatively slow in attacking teeth.

Dr. Canine—One case I had lately, where the tooth was soft and chalky; badly decayed, and having quite a calcareous deposit, why is this? Why was the tartar not dissolved instead of the tooth?

Dr. Cassidy—Because the exciting cause of caries does not run all around hunting something to attack. It attacks at the point of its molecular development, or not at all; if developed in contact with tartar, the latter would suffer to that extent, if developed in contact with the tooth, although tartar be near, it would be only the tooth that would suffer. Were the saliva so generally acid where caries exist, as the stereotyped expression "acid fluids of the mouth" would lead us to believe, there would be no collection of tartar, nor scarcely any teeth.

Dr. H. A. Smith—Explanation of coloring matter not quite satisfactory to me. We have other coloring matter besides the green. I have a patient upon whose teeth is a brownish deposit; it is very hard, and resembles oxide of iron in color. The green-like color is doubtless due to vegetable matter, but I have never been quite ready to believe in the fungoid growth. We do have various coloring matter.

Dr. Cassidy—Yes; chlorophyl is said to be the coloring matter in all vegetables, but they are various in color; so chlorophyl is a misnomer. In the explanation of the cause, or nature, of the green deposit on the teeth of children, I had reference only to that color, and to no other.

Dr. A. Wilkes Smith—I would like to have explained why lead posoning causes a blue line along the margin of the gum.

Dr. Cassidy—Can't say, unless it is due to the presence of lead.

Dr. Edwards—I have in some cases, found the dark line to be due to the use of charcoal as a dentrifice.

Dr. H. A. Smith—I have noticed this blue line very frequently in the Negro race. Would like to know if it is a peculiarity of the African.

Dr. Taft—I suppose the coloring of gum in colored people is due to the same pigment that colors the skin. I know that persons sometimes have this blue line from other causes than lead poisoning. Why it strikes there in lead poisoning, I am unable to say, unless it is the free margin secretes more fluid than the mucous membrane, thus causing deposition of lead.

THERAPEUTICS. "Local Action of Remedial Agents upon Diseased Conditions that Cause Absorption of the Alveolar Process and Gums."

Dr. Peabody—I have worked very hard to correct this receding of the gums, and absorption of the alveolus; have used sulphuric acid a good deal to dissolve the deposit and the necrosed part. When the edge of the alveolus is exposed, there is a peculiar sensitiveness that I can't explain. In treating this trouble with sulphuric acid, it is hard to tell just when to stop. I have sometimes met with success, and have often failed. One case in particular, a case of alveolar abscess of inferior molar, nerve dead, no fistulous opening, could not penetrate with broach through canal, pus came up around the tooth, discharging at the margin of the gum. Treated with one part of sulphuric acid to eight parts of water. Thought from reports of proceedings of Eastern societies, that this was rather weak. Dr. Farrar recommends a solution of one to four, but was afraid to try that. After succeeding in bringing the case under apparent control, I reduced my solution about one-fourth. The face was very much swollen, and at one time I feared it would break externally. After treating some time, the swelling continued. About this time, the patient, against my advice, went to the country; while away, she got her feet wet, and when she came to see me again, the swollen condition of the face was aggravated by great pain and redness, treated some time without effect, and fearing the abscess would break on the outside, I extracted. I found the roots came together at the apex, and the septum came between them, found the septum completely honeycombed, and am convinced the sulphuric acid had attacked it and caused this condition. In the East, I understand, they advocate the use of pure sulphuric acid; if this is so, it is very strange.

Dr. Doyle—Perhaps the honeycombed condition of the septum was due to the action of pus, and not to the acid. I think it likely the damage was done before you saw the case.

Dr. Peabody—Where there is disintegration from pus, there is discoloration. The bone in this case, was perfectly white, and not a particle of odor.

Dr. Taft—I have used preparations of sulphuric acid since it was first introdnced, but never dared to use it as heroically as we are advised to-night. I use only the aromatic sulphuric acid. It will act on the living tissues and not only cause irritation of the soft parts, but will cause death. I think it very likely the condition of the septum just described was caused by the acid. I consider the aromatic sulphuric acid a very good thing, but it should be used very carefully.

Dr. Peabody—I treated the case heroically because it was a very bad one. I have used sulphuric acid, diluted fifteen of water to one of strong acid, in cases of violent inflammation, and reduced the inflammation. I always avoid diluting the aromatic sulphuric acid.

Dr. Taft—In acute stages of inflammation, I would not use it. When necrosis occurs, the inflammation is no barrier to its use. My mode of application is generally with a syringe, with a small point, place the point down well under the gum, and press until fluid appears at the margin.

Dr. Peabody—I have placed teeth in aromatic sulphuric acid, and could see no action whatever.

Dr. Edwards—I have placed teeth in aromatic sulph acid, and seen considerable action; could scrape the enamel off with my finger nail. I remember a case where all the teeth were in bad condition, considerable salivary calculi, gums loose, and standing off from the teeth, pus discharging; teeth seemed as though imbedded in rubber, press them down they would spring back, septum all gone, made application to give relief for the time, made an appointment for thorough treatment, and removed the debris by an operation. Made frequent applications of aromatic sulphuric acid, and treated systemically with iodide of potash, used the acid until I saw the healthy color of the gums, but I could not find any filling up of the alveolus. Adjourned to nine o'clock next morning.

### THIRD DAY—MORNING SESSION.

Discussion on therapeutics continued.

Dr. Peabody—I heard Dr. Canine speak of a case of tumor he treated with strong sulphuric acid, and cured. Please tell us about it.

Dr. Canine—I inserted a lance through to the alveolus, but the tumor continued to spread, I treated with aromatic sulphuric acid four or five times, but the tumor did not seem to yield, I then made a mop, dipped it in pure sulphuric acid, and inserted in the tumor, going clear through to the bottom, cauterized the whole surface. The tumor sloughed out, and the part healed without another application. I saw the patient about a year afterward, entirely well.

Dr. Peabody—I have a case in view which I have not yet examined thoroughly, have not commenced treatment. The first and second superior bicuspids are both dead, and have been filled with some plastic material. The first molar has been extracted. Over the space where the molar was, is an enlargement as big as a walnut, and hard as any portion of the process. There is a constant flow of pus, escaping from the middle of the enlargement. My impression is the flow is from the bicuspids. The tumor is bony, not cartilaginous. The lady is traveling, so I have not been able to begin a systematic course of treatment.

Dr. Taft—If bony and solid cut away the anterior wall, if a cyst, open it freely, the probability is, it is the antrum.

Dr. Doyle—My idea is the trouble comes from those dead bicuspids; the molar probably had an abscess before extraction, and the process became distended; if you cut into it you will find the bone quite cellular. A case in my practice was somewhat similar, an abscessed lateral, though the tooth was perfectly sound. After treatment, the conditions still indicated extraction, which was done. I found the socket spongy. Treated for some days, but pus and soreness continued. I then determined to explore more thoroughly, and soon found my probe passing through a sinus extending to the second bicuspid. The latter had been filled about eight years before. I extracted it at once, and the trouble ceased. I believe, if you open the tumor, you will find a sinus running to the bicuspids, and it won't get well until they are removed. It is best to remove the cause of trouble, even at the sacrifice of a tooth, rather than to permit the tumor to remain.

Dr. Edwards—It would first be best to remove the fillings and see the internal condition of those teeth.

Dr. Peabody—That I intend to do. Dr. Rawls is acquainted with the case.

Dr. Rawls—I consider it a case of diseased antrum, beginning at the second bicuspid, extending to the first, and then gradually involving the whole floor of the antrum, the engorgement of pus distended the walls. I think the case could be cured readily if the patient would submit, but she is timid. I attribute the origin of the trouble to the second bicuspid.

Dr. H. A. Smith—I've long been using sulphuric acid in the treatment of this disease—absorption of the alveoli and gums—I generally prefer to use it strong once, or twice, and then quit it.

Dr. Cassidy—Dr. Smith's heroic treatment, I believe to be generally the best in this disease; but the question on the programme is the "Action of Remedial Agents upon Diseased conditions that cause absorption," etc. There are different stages of this disease, and different symptoms accompanying it, which require different remedies: and what physiological, chemical or physical change do they produce on the part? That's the question. Sulphuric acid will readily dissolve necrosed bone, and living bone also, but in a less degree, thus establishing a line of demarkation, for sloughing between vitality and death. It acts upon the soft parts by dehydration and carbonizing. After careful application of strong sulphuric acid, the aromatic sulphuric acid, an excellent stimulant, and then, thank goodness, we have many other remedies to use in different phases of the disease. What is their action? Potassium chlorate has a special refrigerent action on all mucous surfaces; it has been shown that it communicates an arterial color to venous blood, and in this way may clear the part of congestion; if used to excess, however, it will rapidly cause soreness of the gums. Salycilic acid is an excellent antiseptic, destroying the low forms of organic life; and it is also a good solvent, and for the reason of its apparent ability to soften the long bones, it has been discontinued to a considerable extent in systemic treatment.

Tannin and glycerine combined, afford an excellent application to spongy and inflamed gums. The tannin reducing the diameter of the overcharged vessels, and forming innocuous insoluble tannates of the albuminous matter; the glycerine not only serves as a vehicle for the tannin, but it may assist in the cure of some cases, by abstraction of water for which it has a liking.

The action of iodine is well known; experiment has shown that it causes a rapid extension of the white corpuscles into the subcutaneous cellular tissue. In excessive sensibility, we have a good remedy in aconite; it causes a tingling of the part followed by numbness, due, no doubt, to paralysis of the sensory nerves. It is said also to cause some paralysis of the vaso-motor nerves.

Belladonna is another favorite. It reduces sensibility through a paralysing influence on the sensory nerves. and by direct action on the unstriped muscular fibres surrounding the arterioles. It may also assist in curing by its ability to check excessive salivation. Now, as Dr. Smith has asked for 'a general mouthwash" I will give one of my favorites.

℞ Acidi salycilici.

Soda Biboras. aa. ʒj.

Spts. Rect. f ʒjs,

Aqua Rosae q s. ad-f ℥j. M.

Dr. Taft—Thymol is an agent that has taken the place to some extent in my practice, of creosote and carbolic acid; it is as good an antiseptic as either of those preparations, and indeed has succeeded when carbolic acid has failed.

Dr. H. A. Smith—Thymol has been invaluable in my practice, not only as an antiseptic, but as an obtunder of sensitive dentine. Dry the cavity, and wipe out with the thymol, and go right along with the cutting. I have found salycilic acid, to dissolve bone readily, and for that reason, have nearly abandoned its use.

Dr. A. W. Smith—I have made some very successful experiments with a combination of salycilic acid, thymol and tincture stillingia. I have also found iodoform dissolved in ether exceedingly valuable.

Dr. Peabody--My experience with iodoform has not been satisfactory.

Dr. H. A. Smith—There is often a complete cure of those sore teeth by simply relieving them from pressure, cutting them down sufficiently to give them rest.

Dr. Edwards—I wish to verify Dr. Smith's statement, in regard to the solvent action of salycilic acid on bone. I filled a bicuspid with it, and in a year, although the tooth was splendidly developed, complete solution had taken place.

Dr. Taft—I have used salycilic acid in many cases, and have never known of a case when a tooth suffered by it; I have packed root canals with it years ago, and thus far, not the least trouble is manifest, I am not using it however as much as formerly, there are so many other remedies coming into use. We are making rapid advancement in our knowledge of therapeutics, and I want our young men, to form the habit of making thorough examinations of every case.

Dr. Cassidy—I do not think Dr. Taft's practice of filling roots with salycilic acid, will do for general practice. In his hands it may be safe, because the dry acid, packed so thoroughly in the canal as to exclude moisture, could remain intact indefinitely; but we cannot all use it in this way, so skillfully as Dr. Taft, we do not make with it a water tight filling in the canal, and if moisture reaches it, it will dissolve, and when in solution, it will certainly dissolve bone. Its solvent action is exerted on the calcareous portion, although it affects all tissues.

Dr. Rawls—It seems to me that any one who has had experience with salycilic acid, will admit that it will dissolve bone; it will coagulate albumen, and through that medium may affect good or evil, the various other tissues brought under its influence.

